# The effects of urapidil as an alpha receptor blocker on spinal cord ischemia-reperfusion injury in male rats

**DOI:** 10.1186/s12868-026-01032-3

**Published:** 2026-08-01

**Authors:** Hasan Attila Keskin, Zülfükar Kadir Saritaş, Fatma Görücü Özbek, Aziz Bülbül, Yusuf Koç, Beyza Gül Erdoğan, Zeynep Danaci, Başak Demireller, Salih Fehmi Katircioglu

**Affiliations:** 1 Cardiovascular Surgery Department, Health Ministry, Ankara Education and Training Hospital, Hacettepe Mah. Ulucanlar Cad. No:89, Altındağ-Ankara, Turkey; 2 Department of Surgery, Afyon Kocatepe University, Faculty of Veterinary Medicine, A.N.S Campus, Afyonkarahisar, 03200 Turkey; 3 Department of Physiology, Muğla Sıtkı Koçman University, Milas Veterinary Faculty, Muğla, Turkey

**Keywords:** Spinal cord ischemia, Reperfusion injury, Urapidil, Antioxidant, Neuroprotection

## Abstract

**Objectives:**

The present experimental study was designed to evaluate the potential neuroprotective effects of urapidil during the acute phase (24 hours) of spinal cord ischemia–reperfusion injury in a rat model, with a focus on early functional, biochemical, histopathological, and immunohistochemical changes.

**Methods:**

Rats were randomly assigned to four experimental groups (*n* = 6 per group): Control (CR), Sham (SH), Ischemia–Reperfusion (IR), and Ischemia–Reperfusion + Urapidil (IR+URA). Spinal cord ischemia was induced by infrarenal abdominal aortic clamping for 30 minutes followed by reperfusion. Urapidil was administered before ischemia and after reperfusion in the IR+URA group. Motor function was evaluated 24 hours after reperfusion using the Tarlov scoring system, and biochemical, histopathological, and immunohistochemical analyses were performed. Data were analyzed using one-way analysis of variance (ANOVA) followed by Duncan’s multiple comparison test, which was selected due to its greater sensitivity in exploratory studies with small sample sizes. A *p*-value * < 0.05* was considered statistically significant.

**Results:**

Motor function was significantly impaired in the IR group compared with all other groups, whereas the IR+URA group demonstrated preserved motor performance comparable to the CR and SH groups. Urapidil administration significantly increased antioxidant activity, as reflected by elevated glutathione peroxidase levels, and reduced inflammatory markers including TNF-*α* and IL-1*β*. Histopathological and immunohistochemical findings showed marked neuronal degeneration, apoptosis, oxidative stress, and inflammation in the IR group, which were notably attenuated by URA treatment.

**Conclusion:**

In this experimental spinal cord ischemia–reperfusion model, urapidil treatment was associated with improved early motor performance, increased glutathione peroxidase (GPx) activity, and reduced levels of the pro-inflammatory cytokines TNF-*α* and IL-1*β* during the acute reperfusion period. These changes occurred alongside favorable histopathological findings; however, given the short follow-up duration and the limited scope of apoptotic assessment, the results should be considered preliminary and interpreted as hypothesis-generating.

## Introduction

Spinal cord ischemia–reperfusion injury (SCIRI) is a severe and clinically significant consequence of transient interruption and subsequent restoration of spinal cord blood flow, most commonly encountered during major aortic surgical procedures. Despite substantial advances in surgical techniques and perioperative management, spinal cord ischemia remains a leading cause of postoperative neurological morbidity. Consequently, extensive efforts have been directed toward prevention strategies, including surgical reimplantation of segmental arteries, distal aortic perfusion, cerebrospinal fluid (CSF) drainage, hypothermia, total circulatory arrest, and adjunctive pharmacological therapies such as corticosteroids [1–4].

Clinically, SCIRI encompasses a wide spectrum of neurological outcomes, ranging from subclinical histopathological alterations to irreversible deficits such as paraparesis and paraplegia. In high-risk aortic surgeries, postoperative neurological deficits have been reported in approximately 1.3–16% of patients, while the incidence of permanent paraplegia ranges from 0.3% to 6.5%, depending on surgical extent and patient-related risk factors. Pooled analyses suggest an overall permanent spinal cord injury rate of approximately 3–4% across both open and endovascular aortic repair strategies, underscoring the substantial clinical burden of SCIRI [5, 6].

Although SCIRI is predominantly associated with invasive open aortic procedures involving prolonged cross-clamping and interruption of spinal cord perfusion, spinal cord ischemia has also been reported following thoracic endovascular aortic repair (TEVAR). Contemporary studies document an incidence of approximately 2–8%, particularly in patients requiring extensive aortic coverage, those with prior aortic interventions, or compromised collateral circulation [7, 8].

The pathophysiology of SCIRI is complex and multifactorial, involving an initial ischemic insult followed by secondary reperfusion injury. While reperfusion is essential for tissue survival, it paradoxically exacerbates neuronal damage through excessive production of reactive oxygen species, mitochondrial dysfunction, inflammatory cytokine release, and activation of apoptotic signaling pathways. These mechanisms collectively contribute to secondary neuronal and glial injury and impair neurological recovery [9, 10].

At the cellular and molecular levels, oxidative stress plays a central role in ischemia–reperfusion–related tissue injury. Experimental evidence from ischemia-like and cryopreservation-induced stress models has demonstrated that modulation of oxidative balance through antioxidant interventions can significantly attenuate cellular injury under conditions characterized by rapid metabolic arrest and reoxygenation. For instance, caffeic acid supplementation has been shown to improve antioxidant status and cellular integrity in freeze–thaw injury models, which share mechanistic similarities with ischemia–reperfusion processes [11]. Similarly, herbal antioxidant interventions such as Allium sativum extract have demonstrated protective effects against oxidative stress–induced cellular damage in experimental reproductive toxicity models [12].

At the molecular signaling level, oxidative injury during reperfusion has been closely linked to dysregulation of endogenous antioxidant defense systems, including nuclear factor erythroid 2–related factor 2 (Nrf2) signaling and inducible nitric oxide synthase (iNOS) pathways. Modulation of these pathways has been shown to attenuate oxidative damage and restore redox homeostasis in ischemia-like conditions, further supporting their relevance in SCIRI pathogenesis [13].

Given these interconnected mechanisms, effective pharmacological strategies capable of simultaneously targeting oxidative stress, inflammation, and apoptosis remain limited. Urapidil (URA), a vasodilator introduced into clinical practice in 1988, is widely used in the management of hypertension due to its selective *α*1-adrenergic receptor antagonism and central 5-HT1A receptor activation, resulting in reduced vascular tone without reflex tachycardia. Beyond its antihypertensive properties, URA has demonstrated antioxidant and cytoprotective effects in multiple experimental models of ischemia–reperfusion injury, including the lungs, liver, kidneys, intestines, and reproductive organs [14–18].

Supporting this concept, experimental studies—particularly those evaluating URA in combination with resveratrol—have reported neuroprotective effects in cerebral ischemia–reperfusion models, suggesting a potential therapeutic role in mitigating ischemia-related neuronal damage [19]. Moreover, evidence from preservation and freeze–thaw models indicates that antioxidant supplementation, such as glutathione, can significantly improve oxidative stress parameters and cellular viability under ischemia-like conditions [20].

Additional mechanistic support for antioxidant-mediated tissue protection emerges from reproductive system models employing both conventional and advanced therapeutic approaches. In this context, nanoparticle-based interventions, including silver-doped carbon dots, have demonstrated protective biological effects in experimental models of chemically induced tissue injury, highlighting the potential of nanomaterial-mediated strategies to modulate oxidative and inflammatory pathways [21]. Collectively, findings from these ischemia-like oxidative stress models reinforce the concept that modulation of redox balance represents a shared and critical mechanism of cellular protection across different tissues [22].

In light of these considerations, the present experimental study aimed to investigate the effects of urapidil (URA) in a rat model of spinal cord ischemia–reperfusion injury, with a particular focus on functional, biochemical, histopathological, and immunohistochemical outcomes during the acute reperfusion period.

## Materials and methods

In this study, 24 male healthy adult Wistar Albino rats weighing 250*±*20 g were randomly divided into four groups, each containing six rats ($\mathrm{n}=6$). Rats received intramuscular administration of 13 mg/kg xylazine HCL (Xylazinbio, %2, 50 ml Vial, Bioveta, Bioveta, a.s., Komenskeho Czech Republic) and ketamine hydrochloride (Ketasol, %10, 10 ml vial; İnterhas Medical and Chemical Products Industry and Inc. Ankara-Turkey) for anesthesia. No antibiotics were administered either parenterally or enterally during the experiment. For postoperative pain control, meloxicam was administered intramuscularly as a single dose of 2 mg/kg. (Metacam, 20 mg/ml, 100 ml fl, Boehringer Ingelheim ilaç Tic. AŞ.-Şişli-İstanbul-Türkiye). Comparable injection-based experimental approaches, including studies employing intratesticular and intraperitoneal administration of biologically active compounds, have been widely used to investigate dose-dependent tissue responses and toxicological or protective effects in preclinical models, supporting the methodological validity of the injection protocol applied in the present study [23].

All experimental procedures were approved by the Afyon Kocatepe University Animal Experiments Local Ethics Committee (Approval No: 12/09/2023-49533702/80) and were conducted in accordance with national and international guidelines for the care and use of laboratory animals.

- Group 1 Control (CR; $\mathrm{n} = 6$): Animals in this group did not undergo any surgical intervention or receive any pharmacological treatment. Neurological assessments and tissue sampling were performed for comparison with the other groups.
- Group 2 Sham (SH; $\mathrm{n} = 6$): Under general anesthesia, a median laparotomy was performed, and the abdominal aorta was exposed and isolated without applying an aortic clamp. The abdomen was then closed. No pharmacological treatment was administered in this group in order to control for the effects of anesthesia and surgical manipulation.
- Group 3 Ischemia–Reperfusion (IR; $\mathrm{n} = 6$): In this group, ischemia was induced by infrarenal clamping of the abdominal aorta with a bulldog clamp for 30 minutes. At the end of this period, the clamp was removed to allow reperfusion, and the abdomen was closed. No pharmacological treatment was administered before or after the ischemia–reperfusion procedure.
- Group 4 Ischemia–Reperfusion + Urapidil (IR+URA; $\mathrm{n} = 6$): Animals in this group underwent the same ischemia–reperfusion protocol as described for the IR group. In addition, urapidil was administered in a two-dose regimen before ischemia and after reperfusion. An intramuscular dose of 0.5 mg/kg urapidil was administered 1 hour prior to aortic clamping, followed by a second dose of 5 mg/kg administered immediately after reperfusion and the abdomen was closed.

Previous experimental studies have reported a favorable safety profile of urapidil without histopathological or neurotoxic effects in non-ischemic tissues at comparable doses; therefore, a drug-only control group (Sham + URA) was not incorporated into the predefined study design.

### Surgical procedure

Rats were housed under controlled environmental conditions with appropriate humidity (60–70%) and temperature (23 *±* 2 $^{\circ}$C) and maintained on a 12-hour light/dark cycle, with free access to standard food and water. Food was withheld for 12 hours and water for 6 hours before surgery.

Spinal cord ischemia–reperfusion injury was induced using a previously established and well-described experimental model that reliably produces adequate spinal cord tissue damage [24]. Following transperitoneal access, the abdominal aorta was identified and carefully exposed from the origin of the left renal artery to the distal bifurcation, where vascular clamping was applied. The dose of urapidil (URA) was determined based on previous experimental studies by Güler et al., which demonstrated the safety and efficacy of URA in ischemia–reperfusion injury models [14].

Ischemia was induced by infrarenal clamping of the abdominal aorta using a vascular clamp (Aesculap, Tuttlingen, Germany). Successful induction of ischemia was confirmed by the disappearance of femoral artery pulsation during the 30-minute ischemic period, and reperfusion was verified by restoration of femoral artery pulse following clamp removal. The occurrence of paralysis or paraplegia within the first 24 hours after surgery was assessed in all experimental groups.

The 24-hour reperfusion time point was intentionally selected to capture early oxidative, inflammatory, apoptotic, and functional changes characteristic of the acute phase of spinal cord ischemia–reperfusion injury [23–25], during which apoptotic executioner pathways, particularly caspase-3 activation, are prominently engaged [26–29].

Before sacrifice, blood samples were collected for biochemical analysis, and hind limb motor function was evaluated using the Tarlov scoring system, a validated neurological assessment tool for experimental spinal cord ischemia. Subsequently, spinal cord segments from L4 to L6 were harvested and submitted for histopathological evaluation, as these levels contain the principal motor neuron pools responsible for hind limb function and are particularly vulnerable to ischemic injury following infrarenal aortic clamping [30, 31].

### Euthanasia

After a 24-hour reperfusion period, all rats were deeply anesthetized with intraperitoneal thiopental sodium (200 mg/kg) (Nembutal; Oak Pharmaceuticals, Lake Forest, IL) and subsequently euthanized by overdose, in accordance with AVMA guidelines. Similar experimental approaches involving euthanasia followed by tissue harvesting for oxidative stress assessment have been widely employed in preclinical studies evaluating post-intervention redox status in different organ systems, thereby supporting the methodological framework used in the present study [32].

### Source of animals

All rats were certified Wilstar Albino rats obtained from the licensed breeding colony of the Afyon Kocatepe university Experimental Animals Application and Research Center. All procedures were conducted in the same facility.

### Tarlov scoring system

Motor function was assessed using the Tarlov scoring system, a validated and widely accepted neurological scale for experimental models of spinal cord ischemia associated with aortic clamping [28, 29].

This scoring system is particularly suitable for acute ischemia–reperfusion studies, as it reliably differentiates paraplegia, paraparesis, and preserved hind limb motor function during the early post-injury period.

Motor scores were independently evaluated by two observers blinded to group allocation and graded as follows:

- 0, no movement of the lower limbs;
- 1, slight movement without antigravity strength;
- 2, intermittent movement against gravity without standing;
- 3, ability to stand with abnormal posture or gait;
- 4, normal movement and posture.

### Histopathological evaluation

Spinal cord segments from L4 to L6 were harvested for histopathological analysis. These segments were selected because they contain the principal motor neuron pools responsible for hind limb function in rats and represent the spinal levels most vulnerable to ischemic injury following infrarenal aortic clamping [29, 30]. Evaluation of these levels therefore provides optimal **anatomical–functional correlation** with Tarlov motor scores.

Spinal cord samples were preserved in 4% paraformaldehyde and dehydrated with graded ethanol before paraffin embedding. Similar histopathological approaches employing routine staining techniques and evaluation of oxidative stress–related cellular alterations have been widely used in experimental models investigating antioxidant interventions, thereby supporting the methodological framework applied in the present study [33]. Sections (4 *μ*m thick) were stained using hematoxylin-eosin (H&E) and toluidine blue (TB). For TB staining, paraffin was removed, sections were rehydrated, stained with 0.1% toluidine blue for three minutes, rinsed, dehydrated, and cleared in xylene. Neurons with Nissl substance, loose chromatin, and prominent nucleoli were considered normal, while neurons showing shrinkage, Nissl loss, perinuclear space, and nuclear changes (karyolysis, karyorrhexis, pyknosis) were classified as damaged. Neuron counts were performed in six randomly selected fields at *×*20 magnification.

H&E staining followed standard fixation and embedding protocols, with histological evaluation under light microscopy. Similar immunohistochemical approaches for marker quantification in preserved tissues have been widely used in experimental models evaluating oxidative stress and antioxidant interventions, thereby supporting the methodological framework applied in the present study [34]. For immunohistochemistry (IHC), sections were deparaffinized, rehydrated, and subjected to antigen retrieval in citrate buffer (pH 6.0) via microwave. Endogenous peroxidase was inhibited with 3% hydrogen peroxide. After ultraviolet-blocking solution application, primary antibodies were incubated overnight at 4$^{\circ}$C: eNOS (ab300071, Abcam), TNF-alpha (ab220210, Abcam), MMP9 (ab283575, Abcam), Caspase-3 (ab184787, Abcam), IL-1 beta (ab283818, Abcam), and IL-6 (ab208113, Abcam). Detection was performed using an anti-polyvalent HRP secondary antibody kit (Lab Vision Corp., Fremont, CA, USA) and DAB Chromogen kit (Lab Vision Corp., Fremont, CA, USA). Slides were counterstained with Mayer’s hematoxylin and mounted. A blinded pathologist examined all sections using an Eclipse E-600 microscope (Nikon, Tokyo, Japan) and NIS Elements Image Analysis Software (Nikon, Tokyo, Japan) to quantify degenerative changes and immunopositive neurons [35–37].

Immunohistochemical labeling was assessed based on both staining intensity and distribution. The staining intensity was evaluated using the H-score method, in which 0 indicated no staining, 1+ represented weak staining, 2+ corresponded to moderate staining, and 3+ denoted strong staining.

Immunohistochemical staining was semi-quantitatively assessed using the H-score method, which combines staining intensity and the percentage of positive cells. The H-score (0–300) was calculated based on a modified version of the method originally described by Detre et al., a widely accepted approach in immunohistochemical analysis [38].

### Biochemical evaluation

The biochemical parameters, including nitric oxide (NO), glutathione peroxidase (GPx), tumor necrosis factor-alpha (TNF-*α*), nerve growth factor (NGF), malondialdehyde (MDA), and interleukin-6 (IL-6) levels were measured in blood samples. These analyses were performed with the VGt Lambda Scan 200 ELISA device by using commercial kits (Bio-Tech Instrument, Winooski, VT, USA). Similar biochemical assay-based approaches employing commercial kits to quantify oxidative and metabolic stress parameters have been widely used in experimental models, including studies evaluating ammonia-induced stress in bull spermatozoa, thereby supporting the methodological validity of the stress parameter assessment applied in the present study [39]. Similar biochemical approaches for measuring oxidative stress parameters and antioxidant status have been widely applied in experimental models assessing post-intervention redox balance, thereby supporting the methodological framework used in the present study [40].

These analyses were performed with the VGt Lambda Scan 200 ELISA device by using commercial kits (Bio-Tech Instrument, Winooski, VT, USA).

### Statistical analysis

Each experimental group consisted of six animals ($\mathrm{n} = 6$), which is consistent with sample sizes commonly used in exploratory preclinical spinal cord ischemia–reperfusion studies. The present study was designed as a hypothesis-generating investigation, focusing on detecting biologically relevant differences in acute injury markers rather than on definitive effect size estimation. Differences in the mean values of the measured parameters among groups were assessed using one-way analysis of variance (ANOVA). Prior to ANOVA, the normality of continuous variables was assessed using the Shapiro–Wilk test, and homogeneity of variances was evaluated using Levene’s test. A *p*-value * < 0.05* was considered statistically significant. When a significant overall effect was detected, Duncan’s multiple comparison test was applied to identify intergroup differences. Duncan’s test was selected due to its greater sensitivity in detecting differences between group means in studies with small sample sizes and an exploratory design, where avoiding false-negative results is prioritized over strict control of false positives, whereas more conservative post-hoc tests such as Tukey’s HSD are primarily recommended for confirmatory analyses with larger cohorts. All statistical analyses were performed using the SPSS software package (version 26.0).

## Results

When the Tarlov scores, used to evaluate motor functions as one of the main outcomes of the study, were analyzed among the groups, no statistically significant difference was found between the Tarlov scores of the control, sham, and IR+URA groups ($\mathrm{p} > 0.05$). However, the motor functions of the rats in the IR group were significantly lower compared to those in the other groups ($\mathrm{p} < 0.05$) (Table 1).

**Table 1:** Mean Tarlov scores of the groups ($\mathrm{n}=6$), (Mean*±*SD)

Groups	Tarlov Scores
Control	4.00$^{\mathrm{a}}\pm$0.00
Sham	3.83$^{\mathrm{a}}\pm$0.41
**IR**	**0.33**$^{\mathrm{b}}\pm$**0.52**
IR+URA	3.50$^{\mathrm{a}}\pm$0.55
p values	$$ < 0.001^{*}$$

^a^ Groups sharing the same superscript are not significantly different (Duncan, p>0.05)

^b^ Significantly different from groups marked ^a^ (Duncan, p<0.05)

^*^ Statistically significant (p<0.05)

The mean values of the biochemical parameters for the groups are presented in Table 2. In terms of GPX values, a significant increase was observed in the IR+URA group compared to the other three groups ($\mathrm{p} < 0.05$), while no difference was found between the control, sham, and IR groups ($\mathrm{p} > 0.05$). According to the NO and IL-6 results, a decrease was observed in the IR+URA group compared to the IR group; however, these decreases were not statistically significant. TNF-*α* and IL-1*β* values were significantly higher in the IR group compared to the control group ($\mathrm{p} < 0.05$), and a significant decrease was observed with URA administration ($\mathrm{p} < 0.05$). Regarding NGF values, no statistically significant difference was found among the groups ($\mathrm{p} > 0.05$).

**Table 2:** The comparison of biochemical parameters between the groups ($\mathrm{n}=6$) (Mean*±*SD)

Groups	GPX (ng/mL)	NO (*μ*mol/L)	IL-6 (ng/L)	TNF-*α* (ng/L)	MDA (ng/mL)	NGF (ng/L)	IL-1*β* (pg/mL)
Control	32.00$^{\mathrm{b}}\pm$4.60	18.50$^{\mathrm{b}}\pm$1.79	34.27$^{\mathrm{c}}\pm$2.94	63.83$^{\mathrm{b}}\pm$7.17	60.64$^{\mathrm{c}}\pm$14.59	149.66*±*12.34	17.52$^{\mathrm{c}}\pm$1.28
Sham	32.07$^{\mathrm{b}}\pm$5.16	23.63$^{\mathrm{a}}\pm$2.03	37.32$^{\mathrm{b,c}}\pm$3.87	69.17$^{\mathrm{b}}\pm$14.02	76.07$^{\mathrm{a,b}}\pm$5.96	143.38*±*11.52	17.95*±*1.91
IR	29.54$^{\mathrm{b}}\pm$4.09	23.80$^{\mathrm{a}}\pm$3.24	**45.62**$^{\mathrm{a}}\pm$**9.72**	**85.80**$^{\mathrm{a}}\pm$**9.44**	88.06$^{\mathrm{a}}\pm$7.22	149.52*±*26.93	**34.51**$^{\mathrm{a}}\pm$**9.71**
IR+URA	**43.95**$^{\mathrm{a}}\pm$**2.80**	21.06$^{\mathrm{a,b}}\pm$2.71	42.28$^{\mathrm{a,b}}\pm$3.48	**71.75**$^{\mathrm{b}}\pm$**4.92**	74.00$^{\mathrm{b}}\pm$5.12	160.70*±*15.85	**25.39**$^{\mathrm{b}}\pm$**4.43**
p values	0.001*	0.005*	0.019*	0.014*	0.002*	0.508	0.000*

^a,b,c^ Values indicated by different letters in the same column are statistically different (Duncan, p<0.05)

^*^p<0.001 indicates a significant difference among the groups

Comparable stress-related effects on tissue viability have been observed in other biological systems, such as in guinea pig spermatozoa stored at 5 $^{\circ}$C, where extender composition and cold storage conditions significantly influenced cell viability, further illustrating how stressors can impact tissue integrity and related growth factor responses [41].

The mean values of the biochemical parameters for the groups are presented in Table 2. In terms of GPX values, a significant increase was observed in the IR+URA group compared to the other three groups ($\mathrm{p} < 0.05$), while no difference was found between the control, sham, and IR groups ($\mathrm{p} > 0.05$). According to the NO and IL-6 results, the numerical declines observed in the IR+URA group compared to the IR group did not reach statistical significance ($\mathrm{p} > 0.05$). TNF-*α* and IL-1b values were significantly higher in the IR group compared to the control group ($\mathrm{p} < 0.05$), and a significant decrease was observed with URA administration ($\mathrm{p} < 0.05$). Regarding NGF values, no statistically significant difference was found among the groups ($\mathrm{p} > 0.05$).

### Histopathological results

#### Hematoxylin-Eosin (H&E) staining

In the control (CR) and sham (SH) groups, spinal cord anterior horn morphology was largely preserved, characterized by well-formed motor neurons with granular cytoplasm containing intact Nissl substance, regular nuclear contours, and minimal glial activation. These findings are consistent with normal neuronal structure and metabolic integrity.

In contrast, the ischemia–reperfusion (IR) group exhibited prominent pathological alterations predominantly within the gray matter, including loss of Nissl substance in the cytoplasm (chromatolysis), neuronal shrinkage, cytoplasmic vacuolization, condensed and pyknotic nuclei, and an increased number of degenerative and necrotic neurons. In addition, marked proliferation of glial cells was observed, indicating an early reactive gliotic response to ischemic injury.

**Fig. 1:**
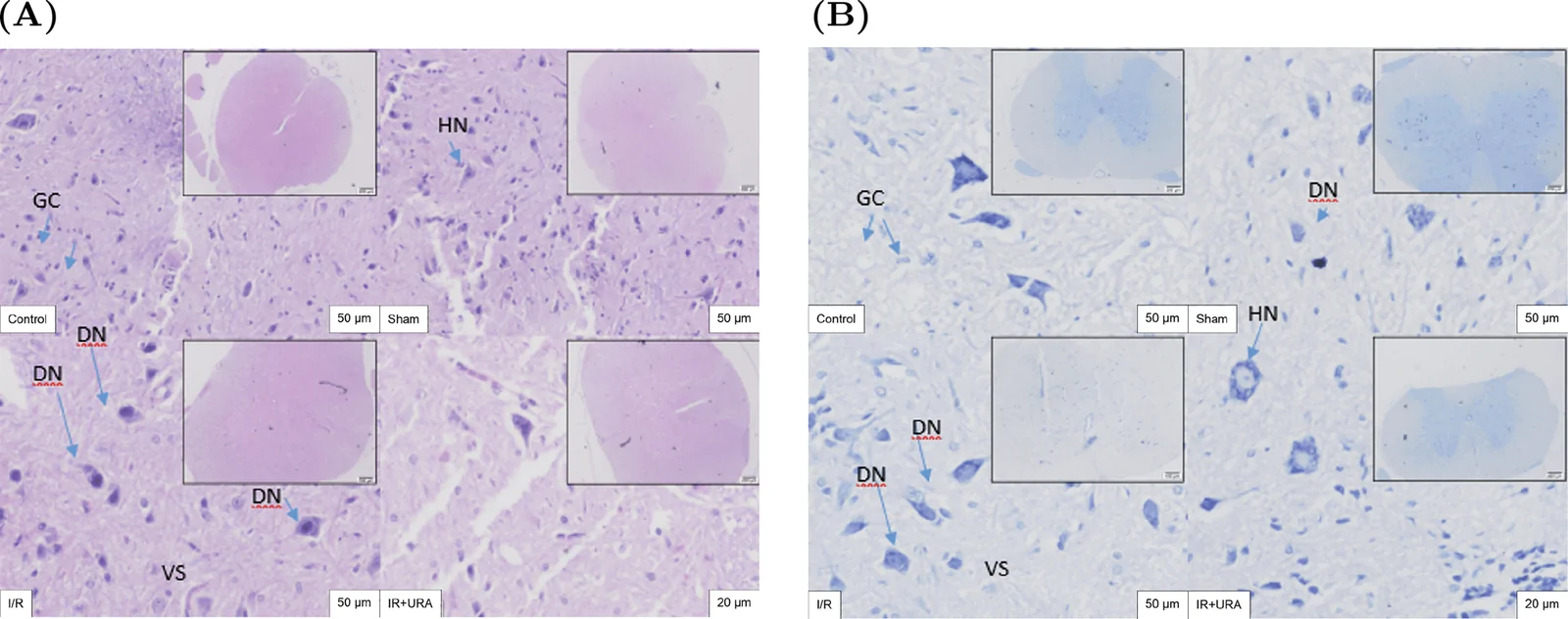
(**A**) Representative histopathological images of the anterior horn of the spinal cord. In the control (CR) and sham (SH) groups, motor neurons largely preserved normal morphology, with only occasional darkened or shrunken neurons and limited glial cell presence. In the ischemia–reperfusion (IR) group, neuronal degeneration and increased glial cell density were more prominent. In the IR + urapidil (URA) group, these histopathological alterations appeared attenuated, with partial preservation of neuronal morphology. GC: glial cell; HN: healthy neuron; DN: degenerated neuron; VS: vascular structure. (**B**) High-magnification histological evaluation of anterior horn motor neurons. In the CR and SH groups, neurons exhibited intact morphology with prominent nucleoli, loose chromatin, and granular cytoplasm. Following ischemia–reperfusion, the IR group demonstrated increased neuronal degeneration accompanied by surrounding glial cell accumulation. In the IR + URA group, neuronal structural features appeared relatively preserved compared with the IR group. GC: glial cell; HN: healthy neuron; DN: degenerated neuron; VS: vascular structure

Treatment with urapidil (IR+URA group) resulted in a partial regression of these pathological changes, with improved preservation of neuronal morphology, reduced chromatolysis, and attenuation of glial proliferation compared with the IR group (Fig. 1A).

#### Toluidine blue (TB) staining

Consistent with the H&E findings, toluidine blue staining demonstrated preserved spinal cord architecture in the CR and SH groups, with well-defined motor neurons exhibiting dense Nissl substance and intact cytoplasmic organization.

In the IR group, TB staining further highlighted extensive disruption of Nissl substance, irregular neuronal outlines, cytoplasmic rarefaction, and pronounced glial cell proliferation within the gray matter, reflecting acute neuronal injury and inflammatory activation following ischemia–reperfusion.

In the IR+URA group, these pathological features were notably attenuated, with improved neuronal staining intensity, partial restoration of Nissl substance, and reduced glial density, supporting a neuroprotective effect of URA at the histological level (Fig. 1B).

### Immunohistochemistry

Caspase-3 activation is one of the irreversible steps in the apoptotic process and is used to investigate changes in apoptosis following ischemic injury and treatment. Positive reactions observed as cytoplasmic staining were minimal in the CR and SH anterior horn samples but significantly increased with IR. This increase was reversed with URA treatment.

#### eNOS immunochemistry

**Fig. 2:**
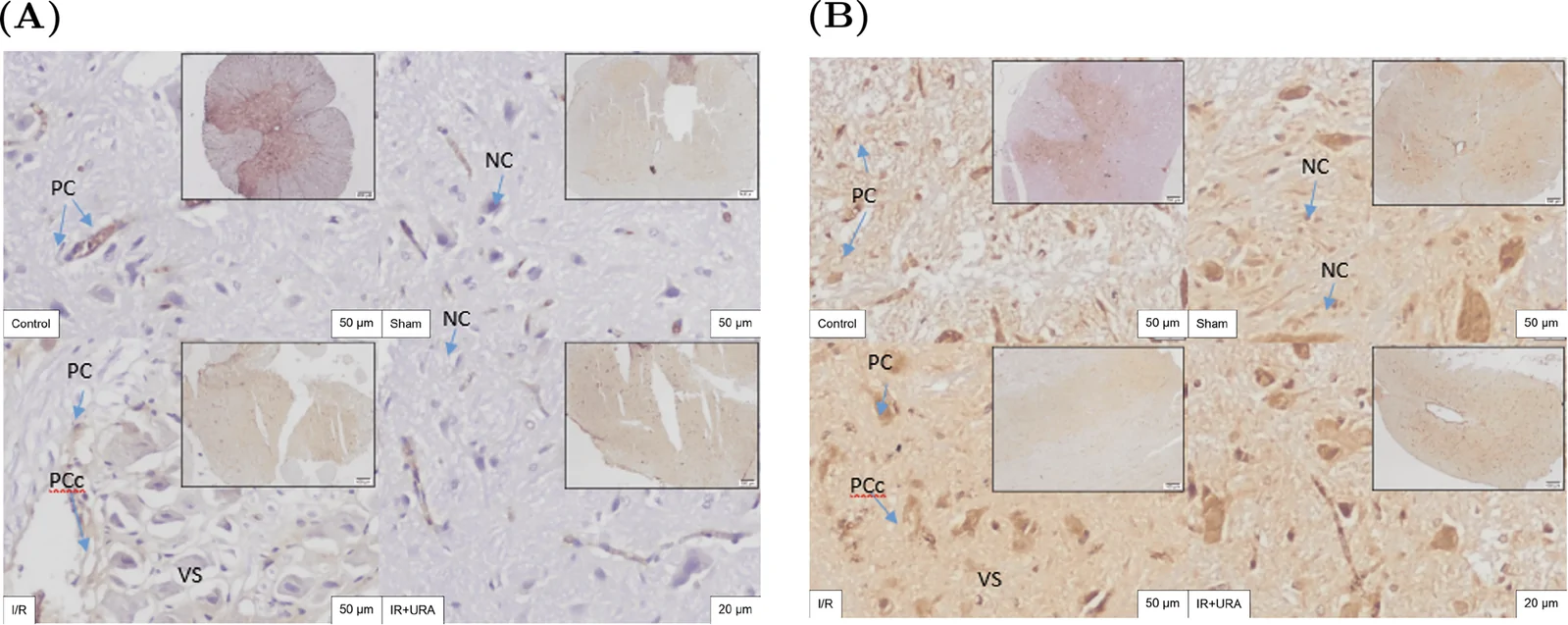
(**A**) Immunohistochemical staining for endothelial nitric oxide synthase (eNOS) in the anterior horn. Compared with the CR and SH groups, altered eNOS immunoreactivity was observed in the IR group. In the IR + URA group, eNOS staining intensity and distribution appeared closer to control levels. PC: positive cell; NC: negative cell. (**B**) Caspase-3 immunohistochemical staining of anterior horn neurons. Minimal caspase-3 immunoreactivity was observed in the CR and SH groups. The IR group showed increased caspase-3–positive cells, indicating enhanced apoptotic signaling. In the IR + URA group, caspase-3 immunoreactivity appeared reduced compared with the IR group. PC: positive cell; NC: negative cell

As a marker of oxidative stress, eNOS IHC was observed to have a basal immunoreactivity in CR and SH anterior horn samples, and this immunoreactivity was increased with IR and decreased with URA (Fig. 2A).

#### Caspase 3 immunohistochemistry

As a marker of apoptotic execution, basal immunoreactivity for caspase-3 was minimal in the control (CR) and sham (SH) anterior horn samples. Caspase-3 was selected as a key executioner caspase representing irreversible activation of the apoptotic cascade; accordingly, changes in caspase-3 immunoreactivity were interpreted as indicative of apoptotic activity rather than upstream signaling events. In contrast, caspase-3 immunoreactivity was markedly increased in the ischemia–reperfusion (IR) group, indicating activation of irreversible apoptotic pathways following ischemia–reperfusion injury. This increase was attenuated in the urapidil-treated (IR+URA) group (Fig. 2B).

In the I/R group, motor neurons exhibit intense cytoplasmic caspase-3 immunoreactivity with pyknotic morphology and prominent vacuolization, consistent with ischemia-induced apoptotic injury. In contrast, the IR+URA group shows markedly reduced staining intensity with preserved neuronal architecture, indicating a protective effect of urapidil. Control and sham groups display only basal/background staining with intact neuronal morphology, serving as reference tissue.

#### TNF-*α* immunohistochemistry

**Fig. 3:**
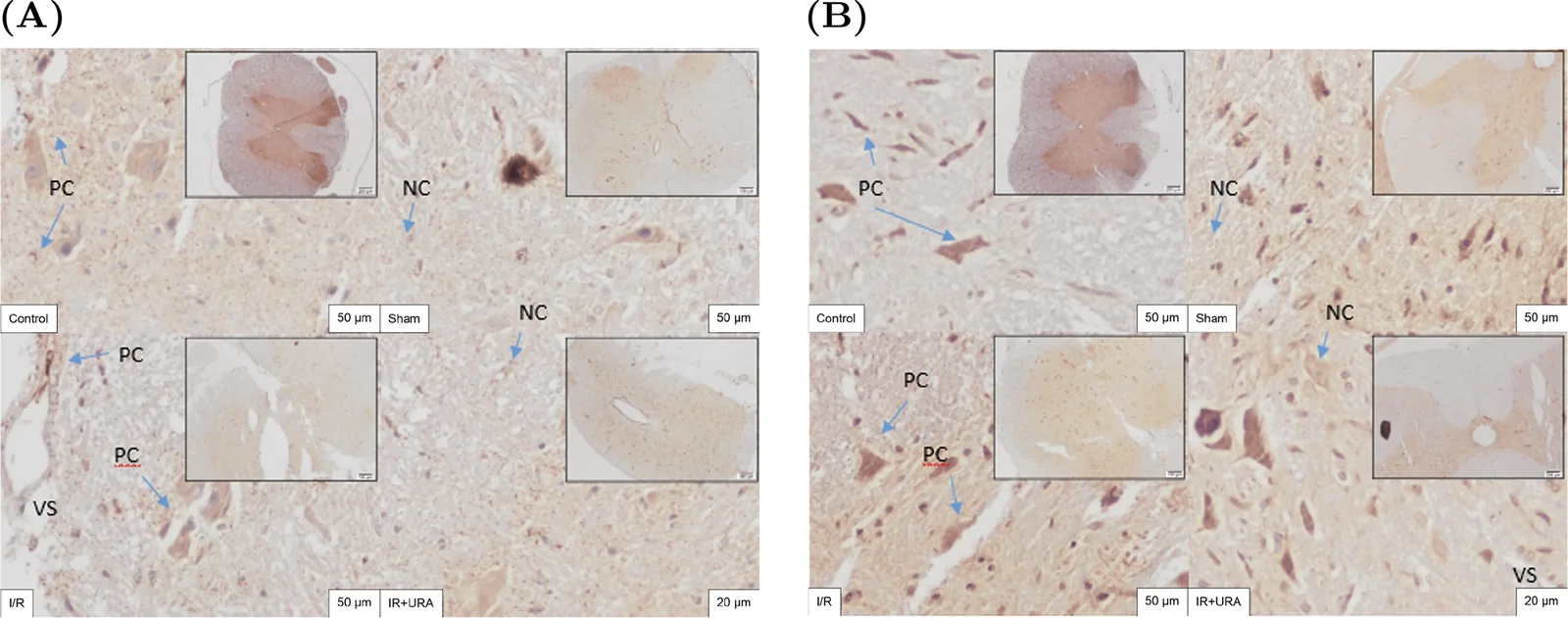
(**A**) Tumor necrosis factor-*α* (TNF-*α*) immunohistochemical staining in the anterior horn. TNF-*α* immunoreactivity was low in the CR and SH groups but was markedly increased in the IR group. In the IR + URA group, TNF-*α*–positive staining appeared attenuated relative to the IR group. PC: positive cell; NC: negative cell. (**B**) Interleukin-1*β* (IL-1*β*) immunohistochemical staining of spinal cord anterior horn tissue. The CR and SH groups exhibited minimal IL-1*β* immunoreactivity. Increased IL-1*β* expression was observed in the IR group, whereas the IR + URA group demonstrated reduced IL-1*β* immunoreactivity compared with the IR group. PC: positive cell; NC: negative cell

For TNF-*α* IHC, it was observed that the basal immunoreactivity in CR and SH anterior horn samples increased with IR and decreased with URA (Fig. 3A).

#### IL-1*β* immunohistochemistry

As an inflammation marker, it was observed that the basal immunoreactivity of IL-1*β* IHC in CR and SH groups increased with IR and decreased with URA (Fig. 3B).

#### IL-6 immunohistochemistry

**Fig. 4:**
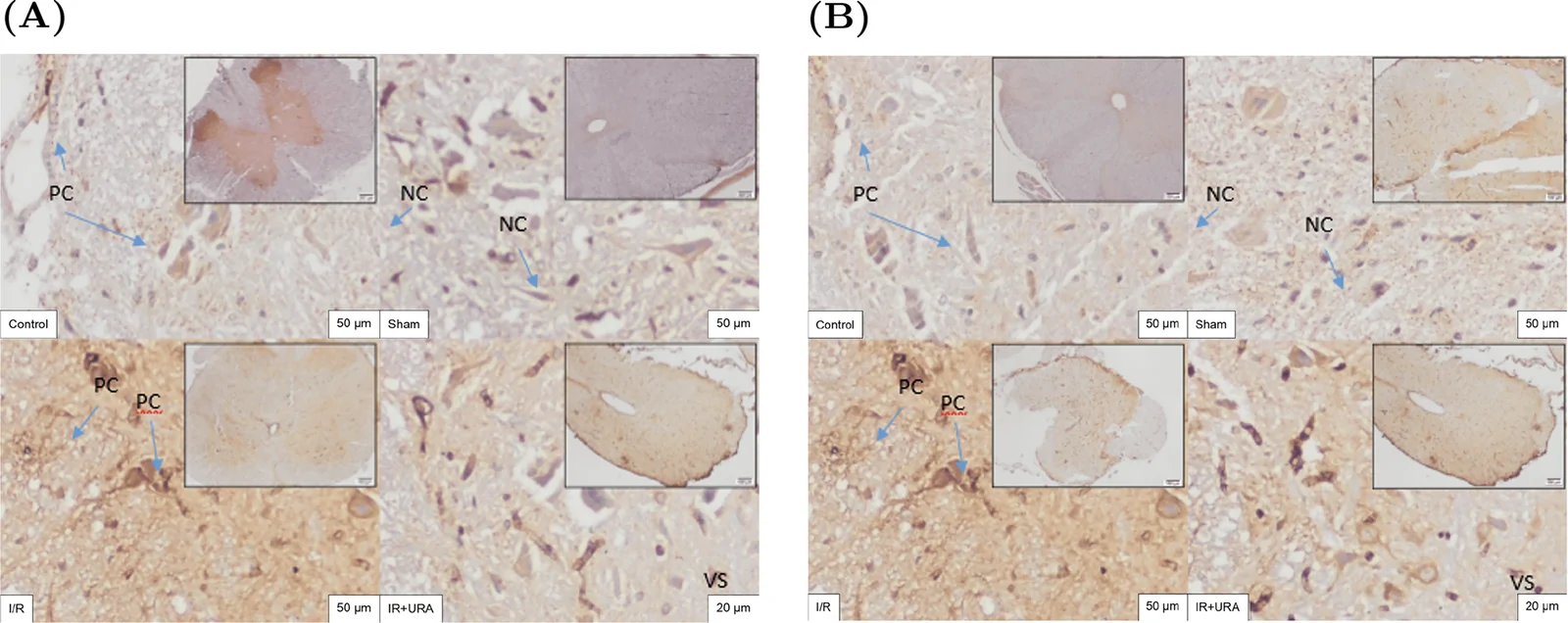
(**A**) Interleukin-6 (IL-6) immunohistochemical staining in the anterior horn region. IL-6 immunoreactivity was minimal in the CR and SH groups and increased following ischemia–reperfusion in the IR group. In the IR + URA group, IL-6 staining appeared less pronounced compared with the IR group. PC: positive cell; NC: negative cell. (**B**) Matrix metalloproteinase-9 (MMP-9) immunohistochemical staining of anterior horn tissue. The CR and SH groups showed weak MMP-9 immunoreactivity, while the IR group exhibited increased MMP-9–positive staining. In the IR + URA group, MMP-9 immunoreactivity appeared reduced relative to the IR group, suggesting attenuation of ischemia-related tissue remodeling. PC: positive cell; NC: negative cell

As an inflammation marker, the basal immunoreactivity present in the CR and SH anterior horn samples was found to increase with IR and regress with URA treatment (Fig. 4A).

#### MMP 9 immunohistochemistry

For MMP 9 IHC, it was observed that a basal immunoreactivity in CR and SH anterior horn samples increased with IR and decreased with URA (Fig. 4B).

### H-scoring results

**Table 3:** H-scores of the groups ($\mathrm{n}=6$) (Mean*±*SD)

	Control	Sham	IR	IR+URA
eNOS IHC	60.33$^{\mathrm{c}}\pm$10.67	76.38$^{\mathrm{a}}\pm$18.40	**136.00** $^{\mathrm{c}}\pm$ **9.21**	**107.60** $^{\mathrm{b}}\pm$ **8.25**
Caspase-3 IHC	192.10$^{\mathrm{a}}\pm$12.04	205.50$^{\mathrm{a,b}}\pm$14.81	**263.90** $^{\mathrm{c}}\pm$ **12.72**	**226.40** $^{\mathrm{b}}\pm$ **7.19**
TNF-*α* IHC	173.90$^{\mathrm{a}}\pm$17.46	188.50$^{\mathrm{a}}\pm$15.84	**252.40** $^{\mathrm{c}}\pm$ **11.36**	**222.60** $^{\mathrm{b}}\pm$ **3.75**
IL-1*β* IHC	158.10$^{\mathrm{a}}\pm$14.22	163.40$^{\mathrm{a}}\pm$15.19	**215.60** $^{\mathrm{b}}\pm$ **8.95**	**183.80** $^{\mathrm{a}}\pm$ **9.28**
IL-6	163.90$^{\mathrm{a}}\pm$9.17	171.90$^{\mathrm{a}}\pm$10.52	**238.00** $^{\mathrm{c}}\pm$ **15.44**	**210.90** $^{\mathrm{b}}\pm$ **7.92**
MMP-9	124.80$^{\mathrm{a}}\pm$22.07	125.50$^{\mathrm{a}}\pm$15.69	**229.00** $^{\mathrm{c}}\pm$ **13.10**	**188.60** $^{\mathrm{b}}\pm$ **10.84**

H&E: Hematoxylin and Eosin; TB: Toluidine Blue; IHC: Immunohistochemistry; eNOS: Endothelial Nitric Oxide Synthase; TNF: Tumor Necrosis Factor; IL: Interleukin; MMP: Matrix metalloproteinase; IR: Ischemia Reperfusion; IR+URA: Ischemia Reperfusion + Urapidil

*Values indicated by different letters in the same row are statistically significant ($\mathrm{p} < 0.05$)

Morphological changes observed in H&E, TB, and immunohistochemical staining were statistically evaluated using H-scoring, and the results are presented in Table 3. In general, no statistically significant difference was found between CR and SH groups in terms of h-scoring ($\mathrm{p} > 0.05$ for all staining types). Again, for all staining types, it was found that damage in control and slightly damaged sham group samples increased significantly ($\mathrm{p} < 0.001$) with IR, while it decreased significantly ($\mathrm{p} < 0.05$) with URA treatment compared to the IR group.

These findings indicate that ischemia–reperfusion induces consistent structural and immunohistochemical injury across staining modalities, while urapidil treatment results in partial but significant histopathological improvement, supporting its early neuroprotective effect at the tissue level.

## Discussion

Spinal cord ischemia–reperfusion injury (SCIRI) remains a devastating complication of aortic surgery and is associated with a broad spectrum of neurological outcomes, ranging from subclinical histopathological alterations to irreversible paraplegia [1–4]. Despite advances in surgical techniques and perioperative management, effective pharmacological strategies that reliably prevent spinal cord injury are still limited. The present experimental study was designed to evaluate the early neuroprotective effects of urapidil during the acute phase of spinal cord ischemia–reperfusion injury, focusing on functional, biochemical, histopathological, and immunohistochemical outcomes at 24 hours.

The primary finding of this study is that urapidil administration preserved motor function during the acute phase following ischemia–reperfusion. While animals subjected to ischemia alone exhibited marked motor impairment, rats treated with urapidil demonstrated motor performance comparable to control and sham groups. These findings indicate early functional preservation following ischemia–reperfusion injury.

Spinal cord ischemia–reperfusion injury is a biphasic process consisting of an initial ischemic phase characterized by interruption of blood flow, followed by a reperfusion phase that triggers secondary injury mechanisms. The reperfusion period is associated with excessive production of reactive oxygen species, activation of redox-sensitive transcription factors such as NF-KB, release of pro-inflammatory cytokines, and initiation of apoptotic pathways, all of which substantially contribute to neuronal injury and functional deterioration [9, 10, 14].

Outcomes were evaluated at the 24-hour reperfusion time point, which was intentionally selected to capture early oxidative, inflammatory, and apoptotic processes characteristic of the acute phase of spinal cord ischemia–reperfusion injury. Previous experimental studies have demonstrated that caspase-dependent neuronal apoptosis and pro-inflammatory signaling peak within the first 24 hours following spinal cord ischemia, whereas later time points predominantly reflect secondary degeneration and functional remodeling [23–25, 28, 29]. Accordingly, this time window provides a biologically relevant snapshot for assessing early neuroprotective effects.

Oxidative stress and inflammation are central contributors to the pathophysiology of spinal cord ischemia–reperfusion injury [42]. Excessive production of reactive oxygen species, mitochondrial dysfunction, and activation of inflammatory cascades play key roles in secondary neuronal damage [43]. Although reproductive organ models do not directly replicate spinal cord ischemia–reperfusion injury, studies employing surgically induced ischemia-like oxidative stress in ovarian tissue have demonstrated that antioxidant interventions can significantly attenuate tissue injury, providing complementary mechanistic support for antioxidant-mediated protection under ischemia–reperfusion conditions [44].

Supporting the role of antioxidant reinforcement under metabolically stressful conditions, supplementation with glutathione and trehalose has been shown to improve oxidative stress parameters and cellular integrity in liquid-stored turkey semen, providing cross-model evidence for the protective effects of antioxidant modulation [45]. In parallel, experimental studies have demonstrated that resveratrol exerts direct neuroprotective effects in spinal cord ischemia–reperfusion models, preserving motor function, reducing lipid peroxidation, enhancing endogenous antioxidant enzyme activity, and attenuating inflammatory cytokine release following aortic cross-clamping [46–48]. At the cellular level, resveratrol limits gray matter–predominant neuronal injury by preserving Nissl substance, reducing caspase-dependent apoptotic signaling, and stabilizing mitochondrial function during the acute reperfusion phase [46, 48, 49]. Evidence from cerebral ischemia models further suggests synergistic neuroprotection with combined resveratrol and urapidil treatment, although spinal cord–specific effects should primarily be interpreted based on direct spinal ischemia models [19].

In the present study, urapidil significantly increased glutathione peroxidase activity, indicating enhanced antioxidant defense during the acute reperfusion period. Supporting the relevance of mitochondrial-targeted antioxidant strategies in ischemia–reperfusion injury, experimental studies in testicular models have demonstrated that Mito-TEMPO administration attenuates oxidative stress, preserves mitochondrial integrity, and improves both biochemical and histopathological outcomes following ischemia–reperfusion injury [50]. In parallel, pro-inflammatory cytokines such as TNF-*α* and IL-1*β* were markedly elevated following ischemia–reperfusion but were significantly reduced with urapidil treatment [51], suggesting that attenuation of oxidative stress and inflammatory responses contributes to the early protective effects observed with urapidil administration.

Complementary mechanistic evidence for ischemia–reperfusion–like injury driven by oxidative and inflammatory stress is further provided by injection-based testicular models, in which intratesticular administration of hypertonic mannitol and saline induced oxidative stress, impaired sperm quality, and caused histopathological damage [52]. Although reductions in certain inflammatory markers, such as nitric oxide and interleukin-6, did not reach statistical significance, these directionally consistent trends occurred alongside significant improvements in motor function and histopathological preservation, suggesting biological relevance during the acute phase of injury. Supporting the broader relevance of antioxidant-mediated protection, synergistic antioxidant combinations, including fisetin and quercetin, have been shown to enhance tissue recovery in testicular ischemia–reperfusion models [53], while toxin-based experimental studies demonstrate that agents such as allicin and hesperidin preserve tissue integrity by attenuating oxidative and inflammatory damage [54].

Collectively, although reproductive system models do not directly replicate spinal cord ischemia–reperfusion injury, they provide complementary mechanistic evidence supporting antioxidant-mediated tissue protection under ischemia-like conditions. Experimental studies in ovarian and testicular torsion–detorsion models have demonstrated that urapidil administration attenuates oxidative stress, inflammatory responses, and apoptosis-related tissue injury [14, 15]. Additional support from sperm and semen stress paradigms, including cold storage and freeze–thaw models, indicates that enhancement of antioxidant capacity, such as glutathione supplementation, preserves cellular integrity under metabolically stressful conditions [20, 41]. Together with evidence from mitochondrial-targeted antioxidant and polyphenolic interventions [50, 52, 53, 55], these findings suggest that modulation of oxidative and inflammatory pathways represents a shared protective mechanism across ischemia–reperfusion–like injury models, indirectly supporting the antioxidant-mediated effects observed in spinal cord ischemia–reperfusion injury.

Consistent with this framework, loss of Nissl substance, neuronal shrinkage, and gray matter–predominant degeneration observed in the ischemia–reperfusion group represent well-recognized histopathological features of acute spinal cord ischemia and reflect early metabolic failure and neuronal injury [56–58]. Similar histological preservation following antioxidant intervention has been reported in reproductive system models, including *β*-cryptoxanthin administration in testicular ischemia–reperfusion injury, further supporting the relevance of antioxidant-mediated structural protection [55, 58].

Consistent with these observations, antioxidant interventions in other ischemia–reperfusion models, including tannic acid treatment, have been shown to attenuate histopathological damage and oxidative stress, providing complementary support for antioxidant-mediated tissue protection [59].

The concomitant increase in glial cell density is consistent with early reactive gliosis, which represents a hallmark of inflammatory activation following spinal cord ischemia and contributes to secondary tissue injury through cytokine release and amplification of oxidative stress [60]. In the present study, urapidil treatment resulted in partial preservation of neuronal morphology, attenuation of chromatolysis, and reduced glial proliferation. These histological improvements are in line with enhanced motor function and modulation of oxidative and inflammatory markers, suggesting that urapidil may exert neuroprotective effects by stabilizing neuronal metabolism and attenuating early secondary inflammatory responses rather than completely suppressing ischemia–reperfusion injury cascades. Importantly, these effects were observed within the acute 24-hour reperfusion window, underscoring early-stage tissue protection, which is particularly relevant in the context of aortic surgery–related spinal cord ischemia.

Importantly, these effects were observed within the acute 24-hour reperfusion window, underscoring early-stage tissue protection, which is particularly relevant in the context of aortic surgery–related spinal cord ischemia.

Spinal cord ischemia–reperfusion injury may arise in several clinical scenarios, including open aortic surgery, endovascular aortic interventions, and circulatory arrest, and can occur despite the use of established protective strategies such as cerebrospinal fluid drainage. In particular, transient spinal cord hypoperfusion followed by reperfusion—commonly encountered during endovascular procedures or perioperative hemodynamic instability—represents a critical mechanism underlying spinal cord injury [61, 62]. The ischemia–reperfusion model employed in the present study was designed to reproduce this pattern of temporary hypoperfusion and subsequent reperfusion injury, thereby reflecting a clinically realistic scenario rather than an artificial or extreme insult. Within this context, urapidil was evaluated not merely as an experimental intervention, but as a pharmacological agent tested against pathophysiological conditions that closely resemble those encountered in contemporary aortic and endovascular practice. In support of this concept, experimental evidence from cryopreservation-induced oxidative stress models has demonstrated that the use of protective additives, such as L-proline and fulvic acid, can significantly attenuate oxidative damage and preserve cellular integrity, underscoring the broader relevance of antioxidant-based preventive strategies in ischemia-like conditions [63]. Similarly, studies in cryopreservation models have shown that co-supplementation with trehalose, fetuin, and taxifolin during freeze–thaw processes markedly improves cellular viability, membrane stability, and antioxidant capacity, providing relevant scientific context for preventive strategies aimed at limiting oxidative and reperfusion-like injury through pre-emptive cytoprotective modulation [64].

In addition to spinal cord–focused models, experimental evidence from reproductive system studies provides complementary mechanistic insights into ischemia-like tissue injury. Ovarian and reproductive tissue models have consistently demonstrated that oxidative stress–induced apoptotic signaling plays a central role in cellular damage following toxic or ischemia-like insults. Alterations in redox balance and apoptosis-related gene expression have been shown to contribute to tissue injury in these models, highlighting shared downstream pathways of oxidative and apoptotic stress across different organ systems. Although these reproductive models do not directly replicate spinal cord ischemia–reperfusion injury, they support the broader concept that modulation of oxidative stress and apoptotic cascades represents a common protective strategy in ischemia-like conditions [65–67].

Patients undergoing aortic surgery frequently present with cardiovascular and metabolic comorbidities, including hypertension, diabetes mellitus, dyslipidemia, and established vascular disease, all of which may exacerbate ischemia–reperfusion–related spinal cord injury and complicate perioperative hemodynamic management. In this clinically relevant setting, the pharmacological profile of urapidil may offer a translational advantage, as it provides effective blood pressure control without reflex tachycardia or pronounced sympathetic activation. Moreover, evidence suggesting favorable effects of urapidil on glucose and lipid metabolism further supports its potential suitability in patient populations characterized by metabolic comorbidity [68, 69]. Accordingly, comorbidities were discussed not as experimental variables in the present model, but to emphasize the clinical realism and translational relevance of urapidil in contemporary aortic surgery practice.

Although urapidil is primarily classified as an antihypertensive agent, its pharmacodynamic profile does not conflict with neuroprotection; on the contrary, it may confer distinct advantages in the setting of spinal cord ischemia–reperfusion injury. Unlike conventional *α*1-adrenoceptor antagonists, urapidil lowers arterial blood pressure without inducing reflex tachycardia, thereby maintaining hemodynamic stability during the critical reperfusion period. Prevention of abrupt blood pressure fluctuations is particularly relevant, as rapid changes in perfusion pressure can exacerbate secondary spinal cord injury through increased oxidative stress, endothelial dysfunction, and disruption of the microcirculation. In addition, urapidil’s dual mechanism of action—combining peripheral *α*1-adrenoceptor blockade with central 5-HT1A receptor–mediated sympathoinhibition—may further contribute to neuroprotection by reducing sympathetic overactivity, stabilizing spinal cord perfusion, and attenuating excitotoxic, oxidative, and inflammatory cascades [70].

Histopathological and immunohistochemical analyses further supported these functional and biochemical findings. Ischemia–reperfusion resulted in pronounced neuronal degeneration, disruption of Nissl substance, glial proliferation, and increased immunoreactivity for markers of oxidative stress (eNOS), apoptosis (caspase-3), and inflammation (TNF-*α*1, IL-1*β*, IL-6, and MMP-9). Treatment with urapidil substantially reduced these pathological changes.

Although apoptosis was evaluated using caspase-3 immunohistochemistry in the present study, neuronal death following spinal cord ischemia–reperfusion injury is known to involve a complex interplay of necrotic, apoptotic, and autophagic mechanisms. Caspase-3 was therefore selected as a representative downstream effector of apoptosis, providing a reliable indicator of irreversible apoptotic activation during the acute phase rather than a comprehensive assessment of upstream cell death pathways [71, 72].

The observed reduction in caspase-3 immunoreactivity with urapidil treatment may reflect combined effects of improved spinal cord perfusion and centrally mediated neuroprotective modulation, potentially involving attenuation of excitotoxicity, oxidative stress, and inflammatory signaling. Although caspase-3 alone does not fully characterize apoptosis, it represents a well-established final effector of the apoptotic cascade and provides a reliable indicator of early apoptotic activity during the acute reperfusion period [73, 74]. Experimental studies have demonstrated that oxidative and inflammatory stress following spinal cord ischemia is closely associated with caspase-3–mediated neuronal apoptosis [75]. In addition, central 5-HT1A receptor activation has been shown to reduce neuronal death and glial responses after ischemic injury [76]. Together, these findings support a dual-mechanism hypothesis for urapidil-mediated neuroprotection following spinal cord ischemia–reperfusion injury, integrating hemodynamic stabilization with centrally mediated anti-apoptotic modulation [14, 77].

Urapidil is pharmacologically distinct from conventional *α*1-adrenoceptor antagonists due to its dual mode of action, combining peripheral *α*1-adrenoceptor blockade with central 5-HT1A receptor interaction/agonism. Receptor binding and pharmacological studies have demonstrated that urapidil exhibits significant affinity for 5-HT1A receptors within the central nervous system, contributing to sympathoinhibitory and centrally mediated effects beyond peripheral vasodilation [78–80]. In the context of ischemia–reperfusion injury, activation of 5-HT1A receptors has been shown to exert neuroprotective, anti-apoptotic, and anti-inflammatory effects, including attenuation of neuronal death, suppression of glial activation, and modulation of excitotoxic signaling in experimental models of cerebral ischemia. Accordingly, the neuroprotective effects observed in the present study may not solely reflect improved spinal cord perfusion related to *α*1-adrenoceptor blockade, but could also involve direct central cytoprotective mechanisms mediated by 5-HT1A–related signaling pathways.

Nevertheless, as receptor-specific antagonism or downstream signaling analyses were not performed in the present study, this mechanistic interpretation remains hypothesis-generating and warrants cautious interpretation.

Taken together, these findings provide a coherent mechanistic and functional framework for understanding the early neuroprotective effects of urapidil in spinal cord ischemia–reperfusion injury and support further investigation of this agent in experimental models of spinal cord protection.

### Limitations

This study has several limitations that should be acknowledged. First, evaluations were limited to a single acute time point (24 hours), which precludes assessment of delayed neuronal death and long-term functional recovery following spinal cord ischemia–reperfusion injury. Second, although the absence of a Sham + urapidil group represents a limitation, this group was deliberately not included because the study was specifically designed to investigate ischemia–reperfusion–related neuroprotection, and existing experimental literature indicates that urapidil does not exert adverse neurological or histopathological effects in non-ischemic tissues at comparable doses. Although the study was intentionally designed to investigate ischemia–reperfusion–related neuroprotection and existing experimental and clinical literature indicates that urapidil does not exert adverse neurological or histopathological effects in healthy tissues at comparable doses, inclusion of such a group in future studies would provide additional mechanistic insight. Third, the relatively small sample size ($\mathrm{n} = 6$ per group) may limit statistical power and the generalizability of the findings (exploratory), particularly for detecting subtle biochemical differences. Additionally, the exclusive use of male rats represents a limitation in terms of generalizability, as sex-dependent differences in ischemia–reperfusion injury and pharmacological responsiveness were not assessed; female animals were intentionally excluded to avoid potential confounding effects of hormonal fluctuations on acute ischemic and inflammatory responses, and gender-specific experimental models such as chemical castration via intra-testicular zinc-doped carbon dots underscore that male-restricted designs may not be directly generalizable to females [81]. In addition, the assessment of multiple biochemical and immunohistochemical parameters increases the potential risk of type I statistical error. To mitigate this limitation, all analyses were interpreted in conjunction with consistent functional and histopathological findings rather than relying on isolated *p*-values alone. The concordance between motor scores, tissue morphology, and molecular markers supports the biological plausibility of the observed effects. Fourth, although histopathological evaluation revealed increased glial cell density consistent with reactive gliosis, glial proliferation was assessed qualitatively using routine staining methods and was not quantified using specific immunohistochemical markers or objective morphometric analysis, which limits precise evaluation of gliosis severity. Fifth, because urapidil exhibits dual pharmacological actions (*α*1-adrenergic antagonism and central 5-HT1A receptor agonism), and no receptor-specific antagonists or pathway-selective interventions were employed, the present study cannot determine the relative dominance of these mechanisms in mediating the observed neuroprotective effects. Finally, a limitation of the present study is that apoptosis was assessed using caspase-3 immunohistochemistry alone, without inclusion of complementary upstream or confirmatory apoptotic markers such as TUNEL staining, Bax/Bcl-2 expression, or cleaved caspase-3 Western blot analysis. Caspase-3 was deliberately selected because it represents a final common execution pathway in apoptosis and provides a robust indicator of irreversible apoptotic activation in acute ischemia–reperfusion injury. Nevertheless, the use of a single apoptosis marker limits mechanistic depth, and future studies incorporating multi-level apoptotic assessment are warranted to further delineate the anti-apoptotic mechanisms of urapidil. In addition, given the exploratory and hypothesis-generating nature of the study, no formal a priori or post hoc power calculation was performed, and this should be considered when interpreting the statistical robustness and generalizability of the findings. Accordingly, the present findings should be interpreted as hypothesis-generating, highlighting early neuroprotective effects rather than definitive long-term neuroprotection.

## Conclusion

The present experimental study suggests that urapidil may exert early neuroprotective effects following spinal cord ischemia–reperfusion injury, as reflected by preservation of motor function and attenuation of oxidative stress, inflammatory responses, and apoptosis-related signaling during the acute reperfusion phase. Histopathological and immunohistochemical findings were consistent with reduced ischemia–reperfusion–related tissue injury; however, apoptosis was evaluated indirectly through caspase-3 immunoreactivity rather than by confirmatory methods.

Given the single 24-hour time-point assessment, the relatively small sample size, the exclusive use of male animals, and the semi-quantitative nature of apoptotic evaluation, the present findings should be regarded as hypothesis-generating rather than indicative of definitive or long-term neuroprotection. Further experimental studies incorporating extended follow-up periods, additional apoptotic and mechanistic analyses, and expanded experimental designs are warranted to clarify the durability, underlying mechanisms, and potential translational relevance of urapidil-mediated spinal cord protection.

## Data Availability

No datasets were generated or analysed during the current study.

## Abbreviations

URA: Urapidil
IR: Ischemia–Reperfusion
SCIRI: Spinal cord ischemia–reperfusion injury
GPx: Glutathione peroxidase
TNF-*α*: Tumor necrosis factor alpha
IL-6: Interleukin-6
IL-1: *β* Interleukin-1 beta
NGF: Nerve growth factor
MDA: Malondialdehyde
eNOS: Endothelial nitric oxide synthase
MMP-9: Matrix metalloproteinase-9
H&E: Hematoxylin and Eosin
TB: Toluidine Blue
IHC: Immunohistochemistry
ANOVA: Analysis of variance
ARRIVE: Animal Research: Reporting of In Vivo Experiments
SD: Standard deviation
CR: Control
SH: Sham
IR+URA: Ischemia–Reperfusion + Urapidil
